# Socio-demographic and regional disparities in utilization of intermittent preventive treatment for malaria in pregnancy - Nigeria demographic health survey 2013

**DOI:** 10.11604/pamj.supp.2019.32.1.13345

**Published:** 2019-01-25

**Authors:** Olukemi Titilope Olugbade, Olayinka Stephen Ilesanmi, Aishatu Bintu Gubio, Ikeoluwapo Ajayi, Patrick Mboya Nguku, Olufemi Ajumobi

**Affiliations:** 1 Nigeria Field Epidemiology and Laboratory Training Programme, Abuja, Nigeria; 2 Federal Medical Centre, Owo, Ondo State, Nigeria; 3 National Malaria Elimination Programme, Federal Ministry of Health, Abuja, Nigeria; 4 Department of Epidemiology and Medical Statistics, Faculty of Public Health, University of Ibadan, Ibadan, Nigeria

**Keywords:** Malaria, sulphadoxine-pyrimethamine, pregnancy, antenatal clinic, intermittent preventive treatment, Nigeria

## Abstract

**Introduction:**

malaria in pregnancy can lead to medical emergencies. Utilization of intermittent preventive treatment for prevention of malaria in pregnancy with Sulphadoxine- Pyrimethamine (IPTp-SP) has remained low in developing countries in sub-Saharan Africa. This study aimed to identify the factors determining uptake of IPTp-SP in Nigeria using the 2013 Nigeria Demographic Health Survey.

**Methods:**

we conducted a secondary analysis of data extracted from the National Demographic Health Survey 2013 which used a three stage stratified sampling method to select respondents. Independent variables considered were age, marital status, the level of education of respondents and their spouses, region, location of residence, wealth-index and birth order. The dependent variable was the use of two or more doses of SP for IPTp in the two years before the survey. Descriptive statistics for socio-demographic and selected characteristics was done. Chi-square test was used to test associations between sociodemographic characteristics and IPTp-SP uptake. Multiple logistic regressions at 95% confidence interval were used to determine predictors of IPTp utilization using STATA version14 software.

**Results:**

of the 38,948 women interviewed 12,473 (32%) had given birth two years preceding the survey and 15% used at least two doses of SP for IPTp. Women aged 30 years and above [aOR 1.4, C.I:1.1-1.7], in the middle class or higher wealth index [aOR 1.5, CI: 1.1-2.0], with two or more ANC visits [aOR 4.2, CI: 1.4 - 12.5], were more likely to use IPTp.

**Conclusion:**

late initiation of IPTp after the second trimester was a contributory factor for poor SP utilization. Interventions targeted at ensuring pregnant women attend ANC and use of IPTp-SP after quickening should be promoted.

## Introduction

About 3.2 billion people remain at risk of malaria globally. An estimated 214 million new cases of malaria and 438,000 deaths occurred in 2015 alone [1]. Approximately 80% of malaria deaths are mainly in Africa and concentrated in just 15 countries. Weak health systems continue to impede progress in most of these countries [1]. Nearly 110 million clinical cases of malaria are diagnosed each year in Nigeria and malaria contributes up to 25% of infant mortality and 30% of under-5 mortality and overburdens the already-weakened health care system in the country [2]. In areas with high malaria endemicity an estimated 50 million women become pregnant annually and 50% of these women reside in Africa [3]. Malaria in pregnancy (MIP) is an obstetric, social, economic and medical emergency and is a major, preventable cause of maternal morbidity, mortality and poor birth outcomes in sub-Saharan Africa. It is responsible for 11% of maternal deaths, 2-5% of maternal anemia, 8-15% low birthweight infants and 3-8% of infant deaths in Nigeria [3, 4]. Maternal mortality in Nigeria is among the highest in the world with a maternal mortality ratio of 576/ 100,000 live births [5] and national efforts to reduce the high maternal and infant mortality place a high premium on effective control of malaria in pregnancy [5, 6]. The proportion of women attending antenatal care (ANC) clinics and those receiving the first and subsequent doses of SP vary which suggests missed opportunities for intermittent preventive treatment of malaria with Sulphadoxine-Pyrimethamine (IPTp-SP) in pregnancy [1].

The Federal Government of Nigeria in 2004 adopted a prevention of Malaria in Pregnancy intervention, as a component of Focused Antenatal Care (FANC). Current recommended approaches for prevention of MIP are IPTp-SP, use of long lasting insecticidal nets (LLINs) and case management following laboratory diagnosis in line with National Malaria Strategic Plan aimed at reducing the burden of malaria in pregnant women The harmful impact of malaria is most apparent in the first and second pregnancies of most pregnant women, living in areas of relatively stable transmission [2]. Due to the reduced immunity of pregnant women who constitute a high-risk group, their protection especially women living in malaria-endemic countries has been of interest to many National Malaria Control and Elimination Programmes [4, 7, 8].

The use of SP for prevention of MIP is a tested intervention with proven effectiveness. The utilization of SP can be expressed as the proportion of pregnant women who received two to three or more doses of SP during an antenatal visit via a directly observed treatment strategy. One dose each of SP is given after quickening from 16 weeks, at 20-24 weeks, 28-32 weeks and 36 weeks or later (at not less than a month interval between each dose) in pregnancy coinciding with each ANC visit [5, 8]. Although malaria preventive services are offered free in all public health facilities, across Nigeria, utilization has remained low. This is mostly because of poor ANC attendance which is the main channel of delivery of IPTp-SP. In some states in northern Nigeria, ANC attendance is less than 30% and national uptake of IPTp-SP in 2008 was 5% [9]. The 2013 Nigeria Demographic Health Survey (DHS) showed that only 10% of women during their last pregnancy reported up to two or more ANC visits and only 18% of women received ANC in the first trimester of pregnancy. Overall 34% of women interviewed in the survey did not receive ANC [5]. The survey further showed that compared to women in rural areas, women in urban areas were more likely than to have their first ANC visit in the first trimester of pregnancy (23% vs 15%) consequently only 13% and 6% women of reproductive age in urban and rural areas received two or more doses of SP [5]. Previous studies done in Nigeria have shown disparities in malaria prevalence across geo-political zones, residence and variations in uptake of SP for IPTp-SP [6, 7, 10-12]. The objective of our study, therefore, was to identify the factors determining the utilization of IPTp-SP in Nigeria.

## Methods

**Figure 1:**
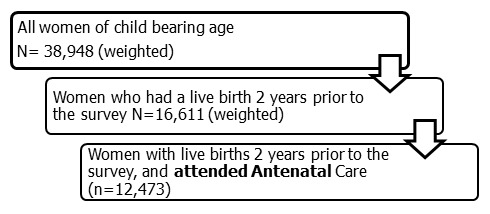
data extraction method from the NDHS 2013

We conducted a secondary data analysis of the 2013 Nigeria DHS [5]. The survey was conducted in 36 States in the six geopolitical regions of the country, with a cluster based household survey study design adopted. All women of reproductive age (age 15-49 years) who were either permanent resident of the households sample or female visitors present in the households on the night before the survey, who were eligible to be interviewed were considered and were coded in the mother's recode data set. The 2013 Nigeria DHS sampling frame had a total of 38,948 women of child bearing age interviewed, of these we further extracted 16,611 women who had a live birth 2 years before the survey, out of which 12,473 had attended ANC; and were considered as the study sample after applying the weighting factor (Figure 1).

The Nigeria DHS 2013 used a stratified sampling technique selection which was performed in three stages. Stratification was achieved by separating each state into urban and rural areas in the survey. In the first stage, 893 localities were selected with proportionate to size allocation, in the second stage; one Enumeration Area was randomly selected from the selected localities. In larger localities with more than 80 clusters, more than one enumeration area was selected. In total, 904 Enumeration areas were selected. In the third stage of selection a fixed number of households (45 households) were selected in every urban and rural cluster, through equal probability systematic sampling. Eligible women were then selected from each household per cluster [5]. We selected women with a live birth in the two years preceding the survey. Our dependent variable was number of women, who had received two or more doses of IPTp-SP one of which was during ANC. The independent variables comprised age groups, the level of education, marital status, occupation, type of residence, region, wealth index, spouse level of education, where ANC was utilized, the number of ANC visits, when SP was first used and the birth order.

### Data analysis

The analysis was done using STATA version 14, with weighting of the sample size done, using the primary sampling unit (PSU), which was the household. Descriptive statistics were done for socio-demographic characteristics. The Chi-square test was used to test associations between socio-demographic characteristics and IPTp-SP uptake. Multiple logistic regression analysis after ruling out confounding was performed to elicit determinants of IPTp-SP uptake at 5% level of significance.

### Ethical considerations

The Nigeria DHS 2013 was coordinated by the Nigerian National Population Commission. Ethical approval for the survey was obtained from the Nigeria National Health Research Ethics Committee (NHREC) with the Assigned Number NHREC/01/01/2007. Informed consent for the survey was obtained from each respondent at the beginning of every individual interview. For this study, the Nigeria DHS 2013 data set and permission for secondary data analysis was granted by ICF International as part of an African regional data analysis workshop. Confidentiality was maintained by the survey interviewers as records were serialized and did not disclose identity of respondents. The accessed data set was kept on a pass worded computer.

## Results

**Figure 2:**
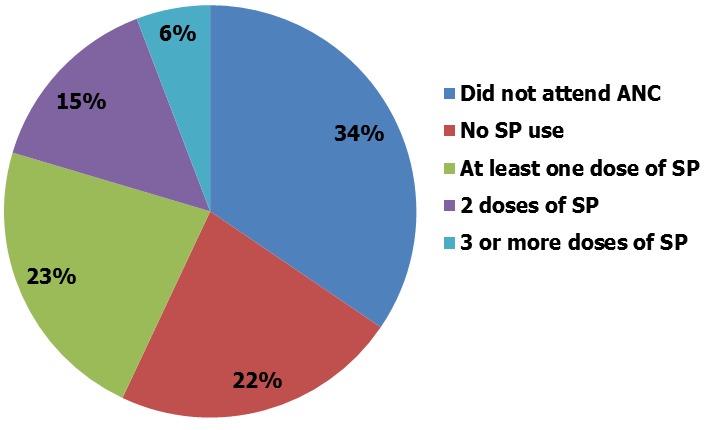
utilization of intermittent preventive treatment using sulphadoxine pyrimethamine by women during pregnancy, Nigeria DHS 2013

Overall 38,948 women of child bearing age were interviewed in the survey. A total of 16,611 (42.6%) had a live birth two years prior to the survey and of these 12,473 (32.0%) women had received SP at ANC (Figure 1). A total 11,573 (92.8%) were married, 7,390 women (59.2%) were in the 15-24 years age range, 5,940 (47.6%) had no form of education and 5,909 (47.4%) were employed. An estimated 4,303 (34.5%) of spouses of the respondents had at least secondary school education or higher. A total of 8,069 (64.7%) of respondents live in rural parts of the country with 854 (6.8%) women in the urban areas utilizing two or more doses of SP. Women in Northcentral, Northwest and Southeast Nigeria, had higher proportion of utilization of SP in their region compared to other regions of the country (Table 1). Overall, 2,869 (23.0%) of interviewed women in the Nigeria DHS sample size (12,473) had taken at least one dose of SP for IPT, 1871 (15.0%) had taken at least two doses of SP and 784 (6.3%) had taken at least three or more doses of SP, with at least one dose, received from ANC (Figure 2).

**Table 1:** sociodemographic characteristics and use of intermittent preventive treatment for malaria by women during pregnancy in Nigeria, Nigeria DHS 2013 (N = 12,473)

	IPTp-SP Utilization			
Characteristics	No SP n (%)	SP 2 or more n (%)	Totaln (%)	p-value
**Age group**				
< 30 years	6,378 (86.3)	1,012 (13.7)	7,390 (59.3)	**0.01**
30+ years		808 (15.9)	5,083 (40.7)	
**Level of Education**	4,275 (84.1)			
None	5,245 (88.3)	695 (11.7)	5,940 (47.6)	**< 0.01**
Primary	1,906 (84.6)	347 (15.5)	2,253 (18.1)	
Secondary/Higher	3,501 (81.8)	779 (18.2)	4,280 (34.3)	
**Marital Status**				
Never	259 (89.9)	29 (10.1)	288 (2.3)	**< 0.01**
Married	9,860 (85.2)	1713 (14.8)	11,573 (92.8)	
Others (divorced, separated, widowed)		79 (12.9)	611 (4.9)	
**Occupation (n = 12,365)**	532 (87.1)			
Unemployed	3,459 (87.4)	499 (12.6)	3,958 (32.0)	**< 0.01**
Employed(unskilled)	5,005 (84.7)	904 (15.3)	5,909 (47.8)	
Employed(skilled)	2,003 (80.2)	495 (19.8)	2,498 (20.2)	
**Type of residence**				
Urban	3,550 (80.6)	854 (19.4)	4,404 (35.3)	**< 0.01**
Rural	7,101 (88)	968 (12)	8,069 (64.7)	
**Region**				
North Central	1,655 (83.1)	37 (16.9)	1,692 (13.6)	**< 0.01**
North East	1,889 (87.8)	262 (12.2)	2,151 (17.3)	
North West	4,635 (83.3)	929 (16.7)	5,564 (36.5)	
South East	940 (81.7)	210 (18.3)	1,150 (9.2)	
South South	1071 (89.9)	120 (10.1)	1,191 (9.6)	
South West	1551 (89.5)	182 (10.5)	1,733 (13.9)	
**Wealth index**				
Poorest	2,706 (93.7)	182 (6.3)	2,888 (23.2)	**< 0.01**
Poorer	2,504 (88.1)	338 (11.9)	2,842 (23.8)	
Middle	1,942 (82.3)	418 (17.7)	2,360 (18.9)	
Richer	1,739 (77.4)	508 (22.6)	2,247 (18.0)	
Richest	1,761 (82.5)	334 (17.5)	2,135 (17.1)	
**Spouse Level of Education (n = 12,065)**				
No education	4,220 (90.6)	438 (9.4)	4,658(38.6)	**< 0.001**
Primary	2,606 (84.0)	497 (16.0)	3,103(25.7)	
Secondary or Higher	3,459 (80.4)	844 (20.0)	4,303(35.7)	

Of the 8172 (65.5%) women who received Antenatal Care (ANC) from government facilities, 1,519 (12.2%) used two or more doses of Sulphadoxine-Pyrimethamine (SP). A total 6372 (51.1%) of the women who attended ANC had three or more ANC visits with only 1,485 (11.9%) of this proportion utilizing two or more SP doses, furthermore 3321 (26.6%) respondents attested to use of SP and up to 2038 (16.3%) of them commenced use of SP in the second trimester of their last delivery preceding the survey (Table 2). Of the women in the middle to richest wealth index 1,260 of them (10.1%) and had used two or more doses of SP for IPTp, while 520 (4.0%) of women in the poorest to poorer wealth index, had uptake of two or more doses of SP.

**Table 2:** antenatal clinic attendance and use of intermittent preventive treatment for malaria by women during pregnancy in Nigeria, Nigeria DHS 2013 (N = 12,473)

	IPTp-SP Utilisation			
Characteristics	No SP n (%)	SP 2 or more n (%)	Total n (%)	p-value
**^+^Type of ANC Clinic (n = 8,172)**				
Government facilities	4,558 (75.0)	1,519 (25.0)	6,078 (74.4)	< 0.001
Private Facilities	1,600 (86.4)	252 (13.6)	1,852 (22.7)	
Others	226 (93.2)	16 (6.9)	242 (3.0)	
**Number of ANC visits (n=8,021)**				
1	245 (97.1)	7 (2.9)	252 (3.1)	< 0.001
2	438 (90.0)	49 (10.0)	487 (6.1)	
3	780 (74.7)	130 (25.3)	910 (11.3)	
>3	4,887 (76.7)	1,485 (23.3)	6372 (79.5)	
**When first SP was first used (n= 3,321)**				
First trimester	185 (44.1)	235 (55.9)	420 (12.6)	< 0.001
Second trimester	803 (39.4)	1,235 (60.6)	2,038 (61.4)	
Third trimester	535 (61.9)	329 (38.1)	864 (26.0)	
**Birth Order (n = 12,473)**				
Less than six	7994 (85.5)	1,356 (14.5)	9,350 (75.0)	0.75
Six and more	2661 (85.2)	462 (14.9)	3123 (25.0)	

+ No ANC use in 4,300 women

Our bivariate analysis showed age > 30years, employment status, having at least secondary school education, marital status, higher level of education of a spouse, skilled employment, urban residence and higher wealth index being associated with higher odds of utilization of two or more doses of IPTp-SP (Tables 2). Independent determinants of IPTp-SP at multivariate analysis were age = 30years [aOR: 1.4, 95% C.I:1.1-1.7], being from the middle to upper class wealth quintiles (aOR: 1.7, 95% CI: 1.1-2.5) or richer (aOR: 2.1, 95% CI: 1.3-3.1)) and having two or more ANC visits (two visits or more visits (aOR: 4.2, 95%CI: 1.4-12.5), three (aOR: 11.4, 95%CI: 4.2-31.5), four or more visits (aOR: 11.6, 95%CI: 4.3-31.4)]. Residents of South-West and [aOR 0.4, CI: 0.2-0.7] and those who commenced IPT in their third trimester of pregnancy [aOR 0.3, CI: 0.2-0.5] were less likely to have received 2 or more doses of IPTp-SP (Table 3).

**Table 3:** determinants of intermittent preventive treatment for malaria by women during pregnancy in Nigeria, Nigeria DHS 2013

Characteristics	OR	95% Confidence Interval (CI)	AOR	95% Confidence Interval (CI)	p-value
**Age (in years)**					
< 30	1.00 (Ref)		1.00 (Ref)		
30+	1.19	1.07 - 1.32	1.36	1.12 - 1.66	0.002
**Level of education**					
No education	1.00 (Ref)		1.00 (Ref)		
Primary	1.37	1.20 - 1.58	0.89	0.67 - 1.17	0.405
Secondary or higher	1.67	1.5 - 1.88	1.06	0.77 - 1.47	0.707
**Marital Status**					
Never Married	1.00 (Ref)		1.00 (Ref)		
Married	1.55	1.05-2.29	1.11	0.65 - 1.90	0.69
Others	1.33	0.85-2.08	0.94	0.48 - 1.84	0.85
**Employment**					
Unemployed	1.00 (Ref)		1.00 (Ref)		
Employed (unskilled)	1.25	1.11 - 1.4	1.03	0.82 - 1.29	0.82
Employed (skilled)	1.7	1.5 - 1.96	0.96	0.75 - 1.24	0.75
**Residence**					
Urban	1.00 (Ref)		1.00(Ref)		
Rural	0.5	0.5 - 0.62	0.99	0.78 - 1.30	0.94
**Region**					
North Central	1.00 (Ref)		1.00 (Ref)		
North East	6.2	4.37 - 8.81	0.59	0.37 - 0.94	0.03
North West	8.97	6.42 - 12.5	1.37	0.87 - 2.16	0.18
South East	9.99	6.98 - 14.3	1.28	0.79 - 2.08	0.32
South South	5.01	3.44 - 7.3	0.83	0.51 - 1.35	0.45
South West	5.24	3.66 - 7.52	0.39	0.24 - 0.65	< 0.001
**Wealth index**					
Poorest	1.00 (Ref)		1.00 (Ref)		
Poorer	2.00	1.66 - 2.42	1.51	1.11 - 2.04	0.008
Middle	3.20	2.66 - 3.85	1.69	1.14 - 2.48	0.008
Richer	4.43	3.63 - 5.20	2.09	1.31 - 3.13	0.002
Richest	2.82	2.33 - 3.41	1.26	0.77 - 2.03	0.35
**ANC utilization**					
Government health facilities	1.00 (Ref)		1.00 (Ref)		
Private facilities	0.47	0.41 - 0.55	0.89	0.65 - 1.22	0.47
Others	0.21	0.13 - 0.35	0.90	0.31 - 2.63	0.85
**Number of ANC visits**					
1	1.00 (Ref)		1.00 (Ref)		
2	3.92	1.75 - 8.78	4.23	1.43 - 12.49	0.009
3	5.83	2.69 - 12.65	11.44	4.16 - 31.46	< 0.001
>3	10.64	5.00 - 22.60	11.58	4.27 - 31.42	< 0.001
**When first SP was used**					
First trimester	1.00 (Ref)		1.00 (Ref)		
Second trimester	1.21	0.98 - 1.50	0.88	0.66 - 1.17	0.38
Third trimester	0.48	0.38 - 0.61	0.32	0.22 - 0.45	< 0.001
**Spouse level of education**					
No education	1.00 (Ref)		1.00 (Ref)		
Primary	1.84	1.60 - 2.11	1.17	0.88 - 1.56	0.27
Secondary or Higher	2.35	2.08 - 2.66	0.97	0.71 - 1.33	0.84

Ref: Reference category

## Discussion

In determining the predictors of IPTp -SP utilization, we found that women aged greater than or equal to 30 years, those living in urban areas and of the middle to upper wealth index with two or more ANC attendance visits had higher odds of IPTp-SP utilization. Utilization of IPTp-SP was low in Nigeria on a national scale. Considering the geo-political zones, sub-national scale utilization was lowest in the northeast, thereafter the southwest and south-south regions of the country. The data showed only 15% of pregnant women interviewed received the recommended two or more doses of SP, with at least one dose administered during an ANC visit, compared to 5% in the 2008 Nigeria DHS [5]. Though there are marginal improvements between both surveys, these are far below the 80% national target for IPTp-SP utilization. Comparing previously and similarly conducted Demographic Health Surveys around the same time frame, the results from Nigeria show similar findings in some Low and Middle income countries (LMICs). Evidence shows low coverage from 5%-17% recorded between 2008 to 2011 in Kenya, Sierra Leone, Madagascar, Zimbabwe and Mozambique, compared to high coverage with ranges of 25%-65% between 2008 to 2011 in Uganda, Cameroon, Tanzania, Burkina Faso, Ghana, and Malawi [13].

The national value from this analysis is lower compared to previous studies done in some states across the country. In rural Ekiti state, Southwest Nigeria 27.3% of ANC attendees of public health facilities, were found to have utilized at least one dose of SP by Falade et al. in 2009 [10]. A study conducted by Amoran et al. in 2012, in Sagamu Ogun state, southwest Nigeria showed 40% of currently pregnant women at the time, used SP for IPTp [3]. In South-south Nigeria Ekpereonne et al. in a study in Calabar, recorded utilization of more than two doses SP for IPTp in 31% of women attending ANC clinics [14]. In Rivers State a 58.4% uptake was estimated, by women who took SP in pregnancy in the community, however only 16.4% took their SP at the health facility directly observed by health workers according to the national guidelines [15]. Public health facilities in Enugu, South East Nigeria were recorded to have provided IPTp services to 31.4% of ANC attendees in public facilities and 65% of attendees in private facilities in 2012 [6]. This coverage was also much lower compared to studies conducted in other countries in West and Sub-Saharan Africa; this is possible because in these countries, community based interventions to improve IPTp use have been instituted [16-19].

The Nigeria DHS 2013 found respondent's age to be a determinant of uptake of SP, as women greater than or equal to 30 years were more likely to adhere to IPTp. Studies conducted in Nigeria, Tanzania, Kenya, and Burkina Faso, showed that younger women were less likely to be available for ANC services, take more than two doses of SP or complete the standard regimen for ANC. A systematic review of factors affecting the delivery, access and use of interventions in selected countries in sub-Saharan Africa however, did not find age a strong determinant of SP uptake [16, 17, 19-21].

Our bivariate analysis showed associations between spousal level of education and uptake of SP, as women with uneducated spouses were less likely to utilize SP. In many settings in Nigeria and Africa, women need spousal permission to leave home to access health care services and take prescribed medication. Women who are educated, employed and have educated spouses, are better disposed to good health seeking behavior in pregnancy [22-24]. Use of SP for IPTp was noted to increase as the spousal level of education advanced, this was similar to findings in other studies which explored this association [15, 21, 23, 25]. Rural-urban disparities exist concerning uptake of SP, the NDHS 2013 showed that though utilization of SP was low amongst the women generally, only 19.4% of women in urban areas used two or more doses of SP for IPT compared to women in urban areas; even though this is similar to other studies in Nigeria, studies conducted in other parts of Africa has shown this to be inconsistent [19, 20, 26]. Respondents' as well as spouses' level of education, marital status, employment status and residence, though corroborated as associated factors in IPTp-SP use in this analysis and other similar studies, were not independent determinants to uptake of more than two doses of SP in this study [16, 20, 25].

The wealth index was found to be a determinant for uptake of two or more doses of SP in this study. The survey showed women in the middle class (poorer to richer wealth quintile) and upper class (richest wealth quintile), were twice likely to use SP in pregnancy than those in the poorest wealth category. Other similar studies consistent with our finding show women of child bearing age who are financially empowered may have better access to malaria preventive interventions with more appropriate health seeking behavior [16, 17].

Antenatal Care (ANC) is the main channel of delivery of IPTp-SP in Nigeria; evidence suggests ANC attendance is still low in certain parts of the country and that pregnant women received SP from other sources than ANC [5, 27]. In our analysis women accessed antenatal services with 2 or more ANC visits were four to 12 times more likely to receive two more doses of SP and complete the regimen. This finding is corroborated by other studies carried out in selected states in Nigeria and other malaria-endemic countries in sub-Saharan Africa where optimal antenatal attendance was associated with high uptake and utilization [14, 18-20, 23, 28]. Late initiation of IPTp after the second trimester was a contributory factor to poor SP utilization. This is a potential threat to the prevention of adverse pregnancy outcomes in both mother and fetus resulting from malaria in pregnancy, as evidence shows the utilization of SP for IPTp has clear superiority over other forms of prophylaxis, with undeniable benefits intra and post-partum [17, 20, 29-32]. Early commencement of ANC and higher number of visits have shown higher IPTp-SP uptake and better pregnancy outcomes [8, 32].

The main limitation of this study is that being an analysis of secondary data, we were not able to assess the knowledge of women interviewed on malaria, MIP and the benefits or barriers to utilization of SP. These benefits or barriers could have been explored using focus group discussions and key informant interviews, but are beyond the scope of the methodology for DHS surveys. The survey was conducted two years after the last delivery with the possibility of recall bias.

## Conclusion

Women aged 30 years and above, those in the middle class or higher wealth index and those with two or more ANC visits were more likely to use IPTp-SP. Late initiation of IPTp after the second trimester was a contributory factor to poor SP utilization. To pregnant women in Nigeria, we recommend consistent ANC attendance with commencement of IPTp-SP after quickening and the promotion of both by health practitioners. The Malaria Elimination Programme and the Ministry of Health at the national and sub-national levels should consider women of reproductive age, who are less than 30 years in Nigeria and those in the regions of the country with poor coverage for IPTp-SP, for community directed and health facility based linkage interventions that will improve uptake of IPTp-SP.

### What is known about this topic

- Malaria is an endemic disease in sub-Saharan Africa and in Nigeria;
- Malaria in Pregnancy (MIP) is a major, preventable cause of maternal morbidity, mortality and poor birth outcomes;
- MIP can be prevented by utilization of Intermittent Preventive treatment with Sulphadoxine-Pyrimethamine in Pregnancy (IPTp-SP).

### What this study adds

- Few studies in Nigeria have looked at community based utilization of IPTp-SP at sub-national and national levels - This study gives information about determinants of IPTp-SP utilization in Nigeria using the 2013 Nigeria Demographic Health Survey (NDHS).

## Competing interests

The authors declare no competing interests.
